# Unveiling temporal and spatial research trends in precision agriculture: A BERTopic text mining approach

**DOI:** 10.1016/j.heliyon.2024.e36808

**Published:** 2024-08-24

**Authors:** Yang Liu, Fanghao Wan

**Affiliations:** a Murdoch University, Australia; b Shenzhen Branch, Guangdong Laboratory for Lingnan Modern Agriculture, Genome Analysis Laboratory of the Ministry of Agriculture and Rural Affairs, Agricultural Genomics Institute at Shenzhen, Chinese Academy of Agricultural Sciences, Shenzhen, 518120, China

**Keywords:** Precision agriculture, Agriculture technology, BERTopic, Text ming, Natural language processing

## Abstract

This study leverages the BERTopic algorithm to analyze the evolution of research within precision agriculture, identifying 37 distinct topics categorized into eight subfields: Data Analysis, IoT, UAVs, Soil and Water Management, Crop and Pest Management, Livestock, Sustainable Agriculture, and Technology Innovation. By employing BERTopic, based on a transformer architecture, this research enhances topic refinement and diversity, distinguishing it from traditional reviews. The findings highlight a significant shift towards IoT innovations, such as security and privacy, reflecting the integration of smart technologies with traditional agricultural practices. Notably, this study introduces a comprehensive popularity index that integrates trend intensity with topic proportion, providing nuanced insights into topic dynamics across countries and journals. The analysis shows that regions with robust research and development, such as the USA and Germany, are advancing in technologies like Machine Learning and IoT, while the diversity in research topics, assessed through information entropy, indicates a varied global research scope. These insights assist scholars and research institutions in selecting research directions and provide newcomers with an understanding of the field's dynamics.

## 1 Introduction

Precision agriculture, a key component of modern agricultural science, integrates advanced information technologies such as the Internet of Things (IoT), big data, and machine learning to enhance agricultural productivity and sustainability [1]. This approach enables real-time monitoring and precise management of crop growth [2], thereby improving yields and quality while minimizing resource waste [3]. Technologies such as drones and satellite imagery facilitate high-precision monitoring, enabling the early detection and treatment of plant diseases and nutritional deficiencies [4]. In the context of global challenges such as climate change and population growth, precision agriculture emerges as a crucial strategy for ensuring food security and promoting sustainable agricultural practices [5].

Recent advancements in the field have led to a significant increase in scientific publications, reflecting the widespread adoption and innovation within precision agriculture [6]. These studies document the evolution from conceptual frameworks to practical applications, providing a comprehensive overview of the field's development and future potential. However, traditional methods of research analysis, such as keyword co-occurrence and citation network analysis, often fall short in capturing the dynamic and complex nature of the field [7]. They typically rely on predefined topic categories, which may overlook emerging trends and nuanced themes [8].

Text mining technologies have enabled researchers to study unstructured databases more effectively, addressing these limitations. Among these advancements, the work of Yu et al. [9] and colleagues stands out for its impactful contributions. Their research demonstrates the potential of these methods across various domains, including artificial intelligence [10], the fuzzy domain [9]; [11], and business management [12]. These methodologies’ innovative applications have garnered recognition for their versatility and cross-disciplinary applicability.

To further advance this field, this study employs BERTopic, a state-of-the-art text mining technique based on transformer architecture. Unlike traditional models, BERTopic does not require predefined topic numbers, offering greater flexibility and accuracy in topic extraction. This approach allows for a more nuanced analysis of unstructured data, such as research abstracts, by identifying latent information and knowledge structures. The study aims to map the topic landscape in precision agriculture, analyze topic distributions across various regions and journals, and trace the evolution of these topics over time. The insights gained from this analysis are intended to guide future research directions and provide a comprehensive understanding of the current state and trajectory of the field.

## 2 Material and methods

**Fig. 1:**
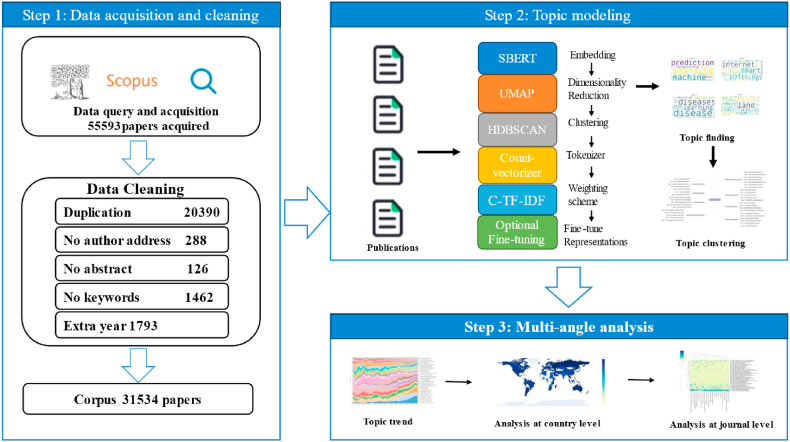
Framework of this research.

The first step involves data downloading and preprocessing. Initially, relevant articles are obtained through keyword searches in the Scopus database. The collected data is then subjected to a data cleaning process, which includes removing duplicate entries, records without author addresses, abstracts, keywords, and any anomalous data. This process results in a refined corpus. The second step involves applying the BERTopic model for topic modeling. This modeling process includes word embedding, dimensionality reduction, clustering, topic representation, and parameter tuning. The third step involves conducting a multi-faceted analysis based on the results of the topic modeling. This includes time series analysis, analysis of topics at the national level, and analysis of topics at the journal level. These are all shown in Fig. 1.

### 2.1 Data source

The primary data for this study were sourced from the Scopus database, a comprehensive repository of peer-reviewed literature. The selection criteria focused on topics, abstracts, and keywords related to “Precision Agriculture,” “Smart Agriculture,” “High-Precision Agriculture,” “Digital Farming,” and “Accurate Agriculture.” The search was limited to English-language articles and conference papers published between January 1, 2000, and December 31, 2023, ensuring a focus on recent research and developments in the field. This process yielded an initial dataset of 55,593 publications. Nevertheless, certain limitations may persist, such as the reliance on specific search terms potentially excluding relevant studies that employ alternative terminology. Despite these considerations, Scopus was selected for its comprehensive and multidisciplinary coverage, providing a robust foundation for constructing our corpus.

#### 2.1.1 Distribution across years of the dataset

**Fig. 2:**
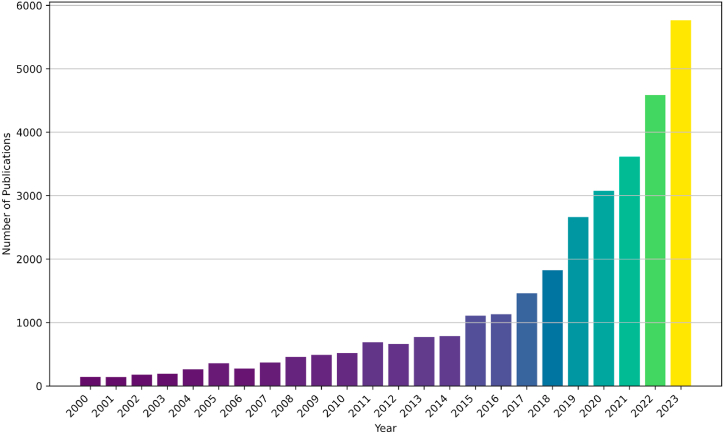
The basic statistical description of Annual distribution of precision agriculture publications.

A year-by-year breakdown of the number of publications highlights the growth and trends in the field, which is shown in Fig. 2.

#### 2.1.2 Geographic distribution

**Fig. 3:**
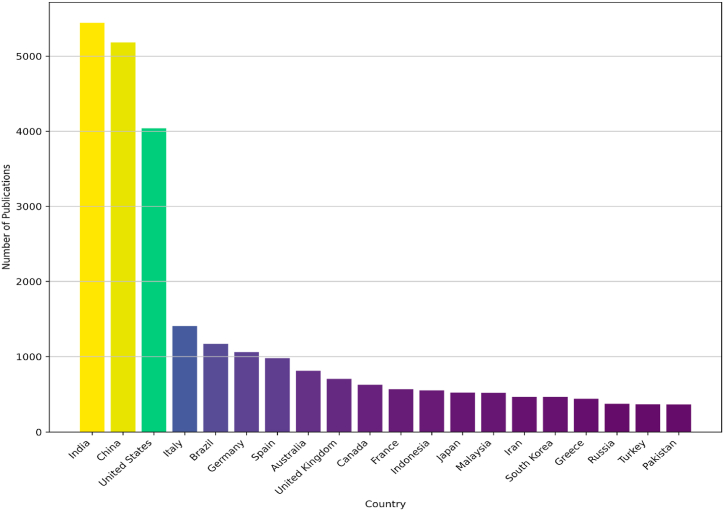
The basic statistical description of top 20 countries publication of precision agriculture publications.

The dataset includes papers from various regions worldwide, illustrating the global engagement in precision agriculture research, which is shown in Fig. 3.

#### 2.1.3 Distribution by subfields

Through the above search restrictions, the following content in Table 1 was obtained and utilized as the original database.

**Table 1:** Precision agriculture related keywords and number of articles.

Search term (Subfield)	Number of papers
Precision Agriculture	17283
Smart Agriculture	10194
Digital Agriculture	8458
High-Precision Agriculture	616
Digital Farming	2437
Smart Farming	4544
Accurate Agriculture	9787
Accurate Farming	2274
Total	55593

### 2.2 Data cleaning

**Fig. 4:**
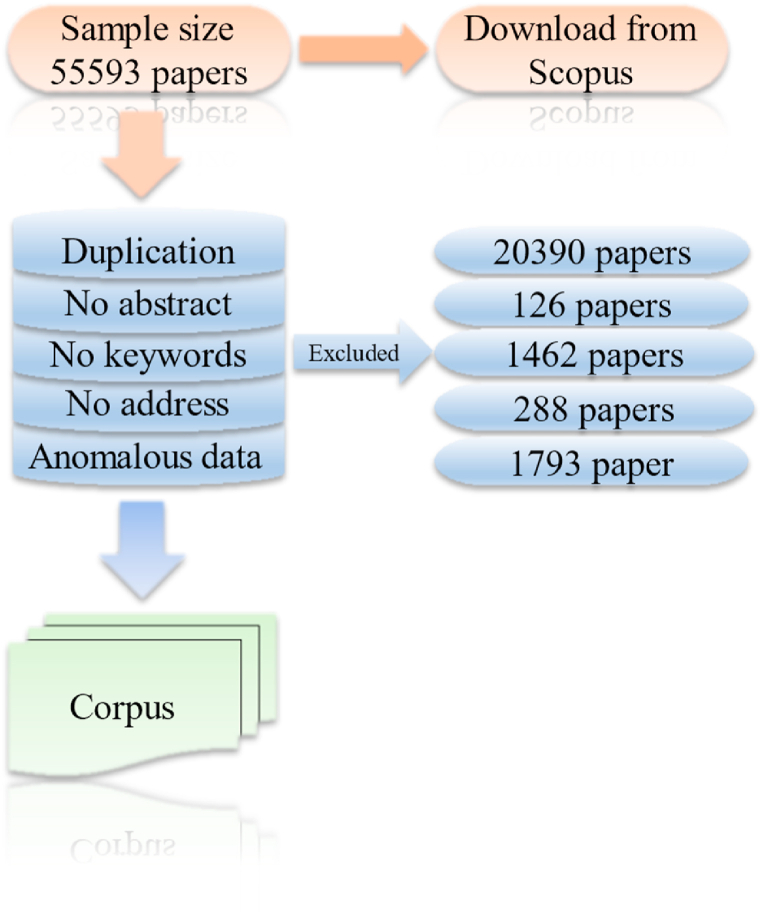
Data acquisition and cleaning process.

To ensure data quality, several preprocessing steps were undertaken, which is shown in Fig. 4. The dataset was first cleansed by removing 20,390 duplicate entries, 288 papers lacking author address information, 126 papers without abstracts, 1462 papers missing keywords, and 1793 papers published outside the specified timeframe. This rigorous cleaning resulted in a refined dataset of 31,534 publications, forming the basis for further analysis. Notably, traditional preprocessing steps like stop word removal were not required due to the use of the BERTopic model, which inherently manages such considerations.

### 2.3 Process of BERTopic modeling

#### 2.3.1 Document embeddings

The algorithm generates document embeddings from a set of documents using either BERT or sentence-transformers in conjunction with pre-trained language models. To extract sentence representations, the “paraphrase-mpnet-base-v2” embedding model was employed within the framework of Sentence-BERT (SBERT) [13]. These embeddings are then compared with previously generated document embeddings using cosine similarity and are labeled accordingly.

#### 2.3.2 Dimensionality reduction

Traditional clustering algorithms often struggle with handling high-dimensional data. To address this, dimensionality reduction is an essential first step before clustering. The UMAP (Uniform Manifold Approximation and Projection) technique, developed by McInnes [14], is commonly employed for this purpose. UMAP is favored for its ability to preserve the structural integrity of the embeddings. Additionally, research by Meng et al. [15] suggests that UMAP outperforms t-SNE in clarity of topic clustering, indicating its superior capability in preserving both local and global data structures.

#### 2.3.3 Document clustering

Following dimensionality reduction, the HDBSCAN (Hierarchical Density-Based Spatial Clustering of Applications with Noise) technique is applied to cluster the embeddings and identify outlier documents. Introduced by McInnes et al. [16], HDBSCAN excels in its ability to handle complex data structures. It clusters documents based on density, facilitating more accurate and meaningful grouping, and enhancing the identification and analysis of distinct topics within the data.

#### 2.3.4 Tokenizer

In this step, text data is tokenized using CountVectorizer. Tokenization is the process of breaking down text into words or phrases. CountVectorizer is a tool used for text feature extraction. It converts text into a matrix of word frequencies, where rows represent samples, columns represent words, and each element in the matrix represents the frequency of the corresponding word in the respective sample.

#### 2.3.5 Topic representation

In contrast to the traditional and time-intensive TF–IDF method, this study utilizes C-TF–IDF (Class-based Term Frequency–Inverse Document Frequency) [17], which operates on the premise that all documents fall under a specific cluster or class. By applying the TF–IDF approach to pinpoint the most significant words within each cluster, we can effectively represent the topics. The concept of C-TF–IDF can be succinctly explained using the following Equation (1):

(1) $$\begin{array}{c} T_{i,c}=tf_{i,c}*\log\left(1+\frac{A}{f_{x}}\right) \end{array}$$

In the data clustering approach, each cluster is distilled into a distinct document, from which the term frequency $tf_{i,c}$ for term i within cluster c is determined. The term $tf_{i,c}$ quantifies the prevalence of term i in cluster c, while A denotes the mean term count for each cluster, and $f_{i}$ encapsulates the frequency of term i throughout all clusters.

#### 2.3.6 Fine-tune topic representation

In this study, the c-TF-IDF methodology was employed to distill a descriptive lexicon that encapsulates the essence of the document collection. This approach not only expedites the generation of precise topic representations but also identifies potential candidates within clusters of keywords and representative documents, which are instrumental for the subsequent refinement of topics. Including representative documents per topic significantly benefits the fine-tuning process on a condensed corpus. By default, BERTopic extracts three quintessential documents per topic. For a more comprehensive analysis to obtain extensive topical content, we fine-tuned the parameters of BERTopic. After fine-tuning and testing, we found that the following parameter settings are most suitable for this dataset.

This study configured the BERTopic model with specific parameters, such as “n_neighbors = 15” and “min_cluster_size = 135.” The “n_neighbors” parameter determines the number of neighbors each point has in the low-dimensional space, thereby affecting the local and global structure of the embedding. A higher “n_neighbors” value focuses more on the global structure, while a lower value focuses more on the local structure [16]. The “min_cluster_size” parameter identifies the minimum number of data points required to form a cluster in the HDBSCAN algorithm. A higher “min_cluster_size” value ensures that clusters are more significant and less susceptible to noise, forming larger and more general clusters. In contrast, a lower value allows the detection of smaller and potentially more specific clusters, albeit at the risk of increased noise [18].

### 2.4 Topic trends

The research content from each year is consolidated from the documents issued within that timeframe. The topic model allocates a document-topic ratio to every document. Consequently, the year-topic ratio can be considered as the average of the document-topic ratios for that particular year.

(2) $$\begin{array}{c} \varphi_{k}^{y}=\frac{\sum_{m\in Y}\varphi_{mt}}{q^{y}} \end{array}$$

$\varphi_{k}^{y}$ represents the proportion of topic t in year y. *m* represents a document published in year *y*. *Y* represents the set of all documents published in year y. $\varphi_{mt}$ represents the proportion of topic t in document m. $\sum_{m\in Y}\varphi_{dk}$ represents the sum of the proportions of topic t in all documents published in year y. $q^{y}$ represents the number of documents published in year y. For example, the time series of topic *k* [$P_{k}^{2000}$, $P_{k}^{2001}$*, …,* $P_{k}^{2023}$]. These time series contain the changing characteristics of the topic distribution and can be employed to discover hot topics.

### 2.5 Topic popularity

In this study, a method based on topic trends and proportions was employed to filter and assess the popularity of topics. The criteria established by Xiong et al. [19] were referenced to evaluate topic popularity, considering both the trend and proportion of topics:

(3) $$\begin{array}{c} P^{k}=S_{NP}^{k}+S_{NTr}^{k} \end{array}$$

where $P^{k}$ denotes the popularity score of topics k, serving as a comprehensive indicator.

(4) $$\begin{array}{c} S_{NP}^{k}=\left(P_{A}^{k}-P_{A}^{\min}\right)÷\left(P_{A}^{\max}-P_{A}^{\min}\right) \end{array}$$

$S_{NP}^{k}$ represents the normalized probability score, where $P_{A}^{k}$ is the average topic proportion of topic k.

(5) $$\begin{array}{c} S_{NTr}^{k}=\left(S_{Tr}^{k}-S_{Tr}^{\min}\right)÷\left(S_{Tr}^{\max}-S_{Tr}^{\min}\right) \end{array}$$

$S_{NTr}^{k}$ is the normalized topic trend score.

(6) $$\begin{array}{c} S_{Tr}^{k}=\frac{\sum_{2013}^{2023}\gamma_{k}^{y}}{\sum_{y=2000}^{2010}\gamma_{k}^{y}} \end{array}$$

$S_{Tr}^{k}$ is the topic trend score.

### 2.6 Topics distribution over countries

This study explores key research themes in different countries and regions, offering insights into the diversity of a specific academic field globally. In this study, each country/region is defined as c, the proportion of topic k is denoted as $\varphi_{k}^{c}$:

(7) $$\begin{array}{c} \varphi_{k}^{c}=\frac{\sum_{d\in c}\varphi_{dk}}{n^{c}} \end{array}$$

In this equation, $\sum_{d\in c}\varphi_{dk}$ represents the total proportion of the $k^{th}$ topic in documents from a specific country or region c, and $n^{c}$ indicates the total number of documents published there. When a document has multiple authors, the country or region of the primary author is used to determine the research's geographical origin.

The metric $\varphi^{c,y}$ represents the annual distribution of topic k across various countries or regions. It is calculated by dividing the sum of the proportional representations of topic k in all documents from country or region c in year y, by the total number of documents published in that country or region during the same year.

(8) $$\begin{array}{c} \varphi_{k}^{c,y}=\frac{\sum_{d\in c\cap d\in y}\varphi_{dk}}{n^{c,y}} \end{array}$$

Here, the numerator $\sum_{d\in c\cap d\in y}\varphi_{dk}$ aggregates the proportions of topic k, reflecting its overall presence in the documents from country c in year y, and $n^{c,y}$ in the denominator accounts for the total document count in country c during year y.

### 2.7 Hot and cold topics over countries/regions

This article conducts both static and dynamic analyses of topic distributions across countries/regions. It employs linear regression on time-series data of topic distributions, resulting in a time-based linear equation for each topic in each country.

(9) $$\begin{array}{c} \varphi_{k}^{c,y}=a*y+b \end{array}$$

Where $\varphi_{k}^{c,y}$ is as defined by Eq (8). The proportion of topic k in year y for each country s and a and b are the slope and intercept of the equation, respectively.

### 2.8 Information entropy of each country

The entropy measure, denoted as $e^{c}$, quantifies the diversity of topics discussed in the research outputs of a country. It is calculated using the equation:

(10) $$\begin{array}{c} e^{c}=-\sum_{k}^{k=1}\varphi_{k}^{c}\;\ln\left(\varphi_{k}^{c}\right) \end{array}$$

where $\varphi_{k}^{c}$ symbolizes the distribution of topic k within the country's research corpus. Higher values of $e^{c}$ suggest a broader array of topics, indicating a diverse academic landscape, whereas lower values suggest more focused research areas.

### 2.9 Topic distribution over publication sources

The content of each publication source is indicative of the topics they explore. Consequently, the method to calculate the topic distribution for any given publication source mirrors the approach outlined in section 2.8. The topic distribution for a publication source s is calculated as follows:

(11) $$\begin{array}{c} \varphi_{k}^{s}=\frac{\sum_{d\in s}\varphi_{dk}}{n^{s}} \end{array}$$

In this equation, $\sum_{d\in s}\varphi_{dk}$ represents the aggregated topic proportions from all documents d published by source s, and $n^{s}$ is the total number of documents released by that publication source. This measure offers a quantitative representation of the prevalence of topic k within the output of source s.

The variable $\varphi^{s,y}$ tracks the yearly distribution of topic k across publication sources s within a specific country, highlighting how the dissemination of this topic fluctuates over time. It is quantified as follows:

(12) $$\begin{array}{c} \varphi_{k}^{s,y}=\frac{\sum_{d\in s\cap d\in y}\varphi_{dk}}{n^{s,y}} \end{array}$$

Here, $\sum_{d\in s\cap d\in y}\gamma_{dk}$ aggregates the proportion of topic k from documents produced by the publication source s during the year y. The denominator, $n^{s,y}$ denotes the total number of documents issued by that source in the same year. This equation allows for a detailed understanding of how the focus on topic k varies annually within each publication source in the country.

### 2.10 BERTopic evaluation

The calculation of Topic Coherence (TC) and Topic Diversity (TD) metrics for topic modeling, specifically using Normalized Pointwise Mutual Information (NPMI), is well-documented in various academic works. The use of NPMI for evaluating topic coherence was notably advanced by Bouma [20], who proposed it as a more reliable measure than traditional PMI because it normalizes the score, making it more interpretable and ensuring it falls within the range of [−1, 1].

For Topic Coherence, NPMI is calculated as follows:

(13) $$\begin{array}{c} PMI\left(\omega_{1},\omega_{2}\right)=\log\frac{P\left(\omega_{1},\omega_{2}\right)}{P\left(\omega_{1}\right)P\left(\omega_{2}\right)} \end{array}$$

(14) $$\begin{array}{c} PMI\left(\omega_{1},\omega_{2}\right)=\frac{PMI\left(\omega_{1},\omega_{2}\right)}{-\log\;P\left(\omega_{1},\omega_{2}\right)} \end{array}$$

The coherence score for a topic is then the average NPMI of all word pairs within the topic. Lau et al. [21] validated NPMI by demonstrating its strong correlation with human judgments of topic quality, leading to its widespread adoption for automated coherence evaluation. Topic Diversity (TD), on the other hand, is defined as the proportion of unique words in the top-k words across all topics. Dieng et al. [22] described it as:

(15) $$\begin{array}{c} Topic\;Diversity=\frac{Number\;of\;unique\;words\;in\;top-k\;words\;of\;all\;topics}{Total\;number\;of\;top-k\;words\;across\;all\;topics} \end{array}$$

Higher diversity indicates less redundancy and more varied topics.

## 3 Results

### 3.1 Topic discovering and clustering

**Fig. 5:**
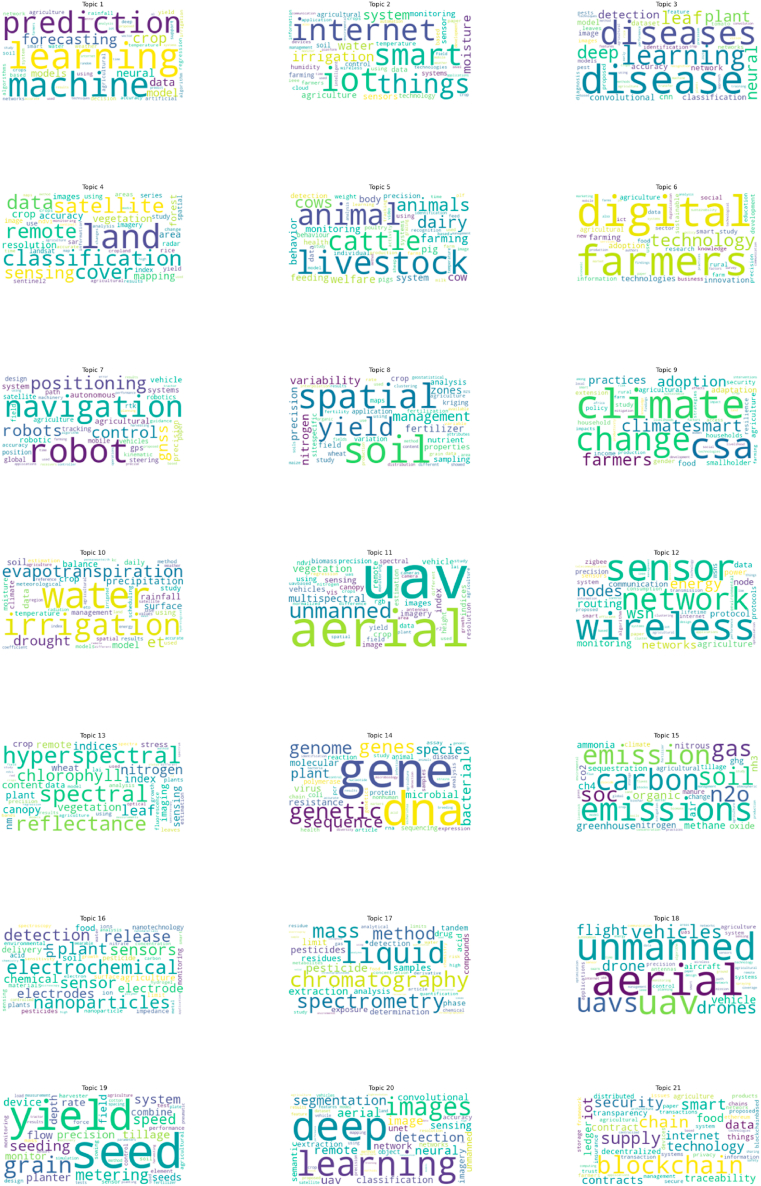

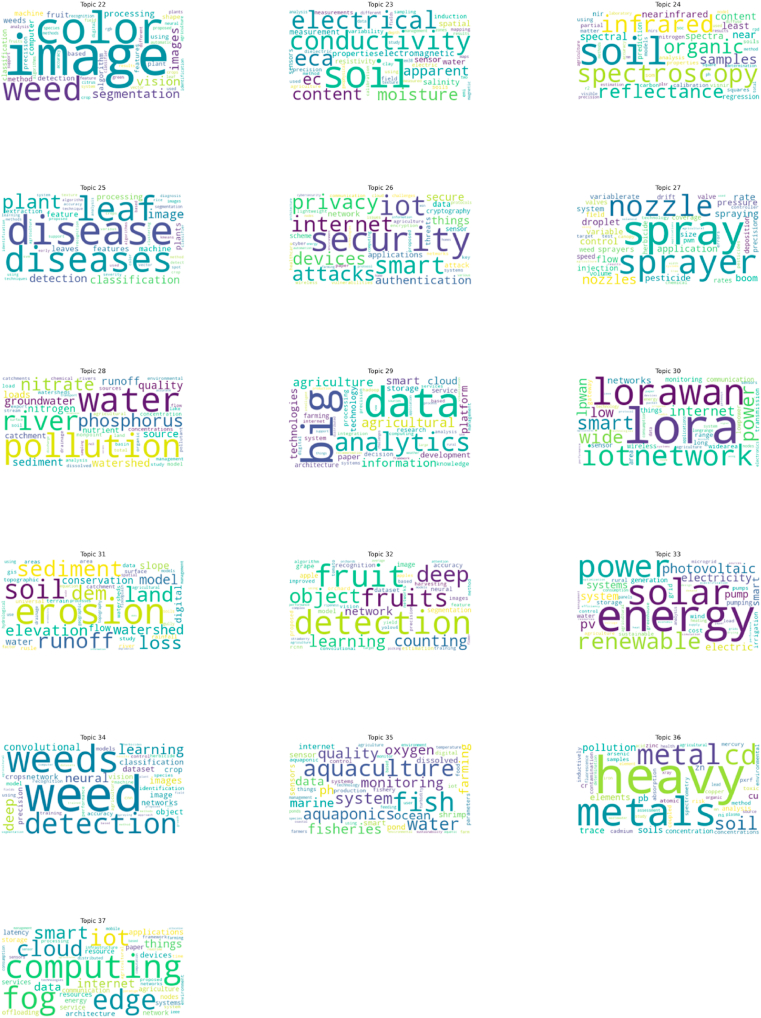
Word clouds depict term frequency within topics, with font size indicating prevalence. Larger fonts highlight words that occur more often across categorized abstracts.

In this study, the BERTopic model was utilized to identify 37 topics, along with their topic-word distributions, using Equation (2). Fig. 5 depicts the relationships between topics and their associated words through word clouds. Each word cloud represents a distinct topic, with its meaning embedded in the words displayed. These word clouds feature the 50 most significant words for each topic, with the size of each word indicating its likelihood of occurrence within the topic.

**Fig. 6:**
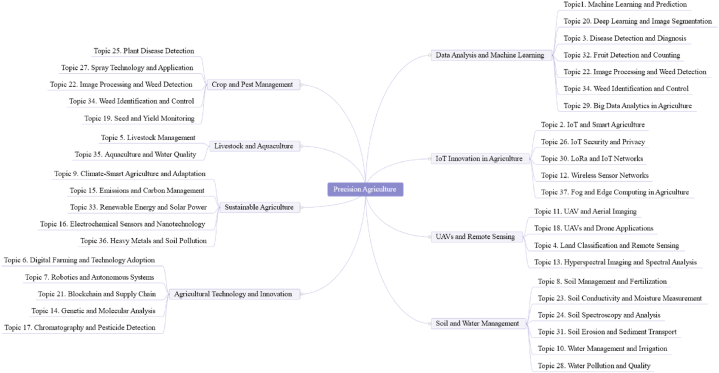
Subfield division of the field of precision agriculture.

These terms in Table 2 are closely related to research in the field of precision agriculture and can be used to define 37 topics as subfields of AI research based on their semantics. For example, Topic 1 consists of terms such as learning, machine, prediction, and forecasting, which are associated with Machine Learning and Prediction (T1). Machine Learning and Prediction is widely applied in the context of precision agriculture for crop yield forecasting, soil analysis, irrigation management, and disease and pest detection. Similarly, all word clouds are defined by their respective themes. From these topics and terms, this study found that words like learning, neural, and data frequently appear across multiple topics. This result indicates that, although there are some differences between these topic terms, they have semantic similarities that allow for clustering topics, thereby constructing higher-level analytical pathways, as illustrated in Fig. 6.

**Table 2:** Summary of the 37 distinct topics with associated keywords and document counts. 19148 articles could not be classified into any specific topic and were therefore omitted.

Topic label	Keywords	Number of documents
1.Machine Learning and Prediction	learning, machine, prediction, forecasting, crop, neural, model, data, models, agriculture	2032
2. IoT and Smart Agriculture	iot, internet, smart, things, irrigation, system, moisture, water, agriculture, sensors	1619
3. Disease Detection and Diagnosis	disease, diseases, learning, deep, leaf, plant, neural, detection, convolutional, classification	1071
4. Land Classification and Remote Sensing	land, classification, satellite, cover, remote, data, sensing, vegetation, crop, mapping	1055
5. Livestock Management	livestock, animal, cattle, animals, dairy, cows, farming, cow, welfare, behavior	1027
6. Digital Farming and Technology Adoption	digital, farmers, technology, technologies, farming, adoption, innovation, agriculture, agricultural, rural	928
7. Robotics and Autonomous Systems	robot, navigation, positioning, robots, control, gnss, agricultural, system, autonomous, systems	896
8. Soil Management and Fertilization	soil, spatial, yield, management, variability, nitrogen, zones, precision, fertilizer, properties	860
9. Climate-Smart Agriculture and Adaptation	climate, csa, change, climatesmart, farmers, adoption, practices, adaptation, food, smallholder	817
10. Water Management and Irrigation	water, irrigation, evapotranspiration, et, drought, precipitation, model, surface, crop, soil	803
11. UAV and Aerial Imaging	uav, aerial, unmanned, multispectral, vegetation, index, sensing, remote, images, vehicles	770
12. Wireless Sensor Networks	wireless, sensor, network, nodes, wsn, energy, networks, routing, monitoring, node	739
13. Hyperspectral Imaging and Spectral Analysis	hyperspectral, spectral, reflectance, chlorophyll, leaf, nitrogen, vegetation, indices, canopy, index	700
14. Genetic and Molecular Analysis	gene, dna, genetic, genes, genome, sequence, species, plant, bacterial, microbial	647
15. Emissions and Carbon Management	emissions, carbon, emission, soil, gas, n2o, soc, organic, greenhouse, ch4	637
16. Electrochemical Sensors and Nanotechnology	electrochemical, nanoparticles, plant, sensor, detection, release, sensors, electrodes, ph, electrode	635
17. Chromatography and Pesticide Detection	liquid, chromatography, spectrometry, mass, method, pesticide, extraction, pesticides, samples, limit	555
18. UAVs and Drone Applications	aerial, unmanned, uav, uavs, vehicles, drones, flight, drone, vehicle, aircraft	508
19. Seed and Yield Monitoring	seed, yield, grain, metering, seeding, speed, system, tillage, precision, flow	443
20. Deep Learning and Image Segmentation	deep, learning, images, segmentation, detection, aerial, neural, remote, image, uav	437
21. Blockchain and Supply Chain	blockchain, supply, chain, smart, security, iot, technology, data, food, contracts	421
22. Image Processing and Weed Detection	image, color, weed, segmentation, vision, images, processing, weeds, fruit, computer	409
23. Soil Conductivity and Moisture Measurement	soil, conductivity, electrical, eca, moisture, content, ec, apparent, electromagnetic, sensor	388
24. Soil Spectroscopy and Analysis	soil, spectroscopy, infrared, organic, reflectance, samples, spectral, near, content, spectra	318
25. Plant Disease Detection	disease, diseases, leaf, plant, image, detection, classification, processing, leaves, features	251
26. IoT Security and Privacy	security, iot, smart, internet, privacy, attacks, devices, authentication, secure, things	232
27. Spray Technology and Application	spray, sprayer, nozzle, nozzles, application, flow, rate, control, spraying, droplet	229
28. Water Pollution and Quality	water, pollution, river, phosphorus, nitrate, groundwater, runoff, quality, watershed,	214
29. Big Data Analytics in Agriculture	big, data, analytics, agricultural, agriculture, information, smart, cloud, platform, technologies	206
30. LoRa and IoT Networks	lora, lorawan, network, iot, smart, power, wide, internet, low, lpwan	193
31. Soil Erosion and Sediment Transport	Erosion, Soil, Sediment, Runoff, Land, DEM (Digital Elevation Model), Loss, Elevation, Watershed, Model	182
32. Fruit Detection and Counting	Detection, Fruit, Fruits, Deep, Object, Learning, Counting, Network, Segmentation, Apple	178
33. Renewable Energy and Solar Power	Energy, Solar, Power, Renewable, Photovoltaic, PV, Electricity, System, Electric, Systems	160
34. Weed Identification and Control	Weed, Weeds, Detection, Learning, Deep, Neural, Convolutional, Images, Classification, Dataset	158
35. Aquaculture and Water Quality	Fish, Aquaculture, Water, System, Aquaponics, Quality, Monitoring, Oxygen, Fisheries, pH	156
36. Heavy Metals and Soil Pollution	Heavy, Metals, Metal, Cd, Soil, Pollution, Soils, Elements, Cu, Pb	150
37. Fog and Edge Computing in Agriculture	Computing, Fog, Edge, IoT (Internet of Things), Cloud, Smart, Internet, Data, Things, Applications	145

### 3.2 Topic trends and prediction in precision agriculture

**Fig. 7:**
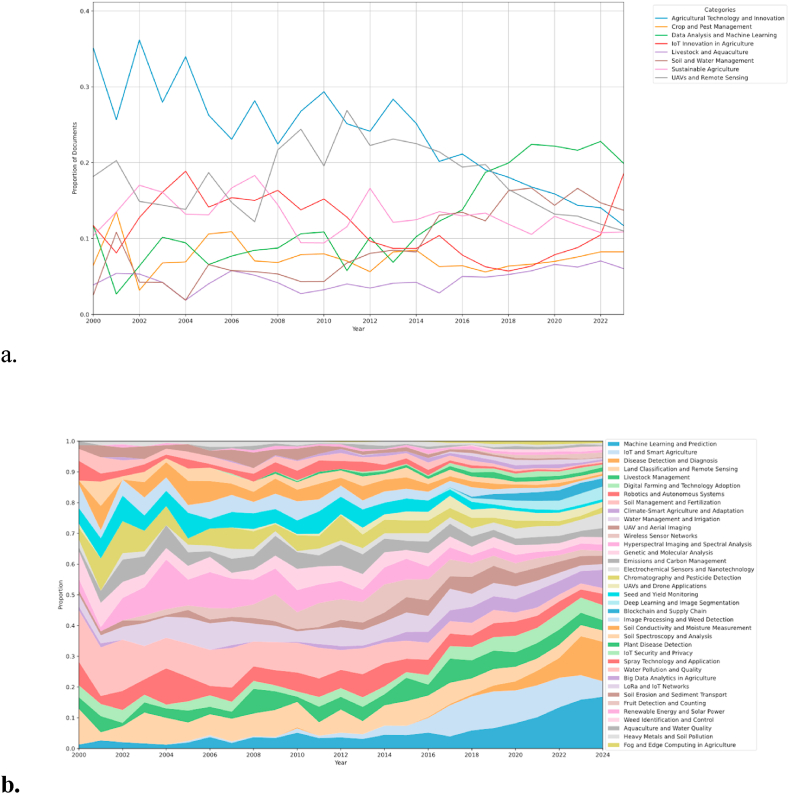
Topic distribution over time in precision agriculture. (a) Line graph showing the trends of 8 subfield topics within precision agriculture from 2000 to 2022. (b) Area plot illustrating the temporal changes in 37 topics within precision agriculture, with the area size reflecting the relative focus on each topic over time.

Fig. 7 shows the distribution of topics and subfields in each year and shows the relative change in popularity over time. Fig. 7(a) demonstrates the subfield topic trends over time, revealing the relative changes in popularity for 8 subfields from 2000 to 2023. In Fig. 7(b), a detailed proportional distribution of 37 topics across these years is depicted.

Agricultural Technology and Innovation has consistently held a significant presence, although it has shown a declining trend over time, starting from around 0.35 in 2000 and decreasing to approximately 0.15 by 2023. In contrast, Crop and Pest Management has seen a steady increase in interest, particularly from 2016 onwards, rising from about 0.05 to just below 0.20 by 2023. Data Analysis and Machine Learning has maintained a relatively stable trend, with minor fluctuations, consistently holding at or below 0.10.

A notable trend is observed in the field of IoT Innovation in Agriculture, which has experienced a marked increase since 2020, rising sharply from below 0.05 to over 0.15 by 2023. This reflects a growing focus on integrating IoT technologies into agricultural practices. The category of Livestock and Aquaculture shows moderate variability, with a slight downward trend, fluctuating around 0.05 over the years. Soil and Water Management has remained relatively stable but low, with slight fluctuations below 0.10 throughout the observed period.

Sustainable Agriculture has gradually gained attention, especially post-2015, increasing from around 0.03 to approximately 0.10 by 2023, indicating a growing awareness of environmentally friendly practices. Lastly, UAVs and Remote Sensing experienced an early peak in the 2000s, followed by a decline and subsequent stabilization around 0.10, suggesting a consistent but not dominant focus in this area. These trends collectively highlight the evolving priorities and technological advancements in precision agriculture over the years.

However, relying solely on the change in topic distribution over time to identify truly popular topics can be misleading. Some topics, despite experiencing a decline in their proportional representation, continue to exhibit relatively high distribution probabilities. Examples of such topics include digital Farming and Technology Adoption (T6), Soil Management and Fertilization (T8), and UAV and Aerial Imaging (T11). These topics should not be hastily deemed unpopular. This study filters the popularity of topics by adopting the criteria proposed by Xiong et al. [19], which comprehensively consider both the trend and proportion of topics.

**Fig. 8:**
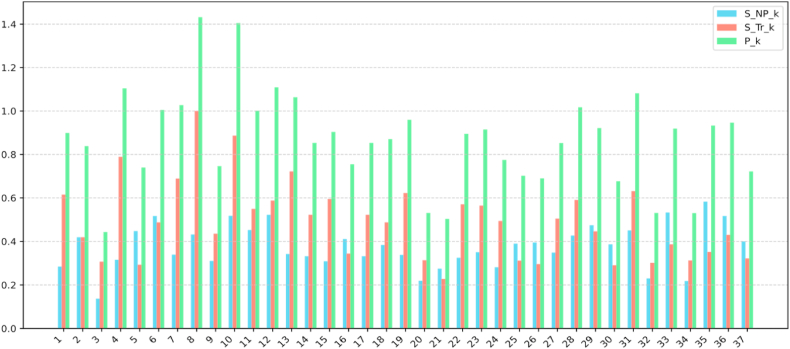
Popularity ranking of topics.

According to Equations (3), (4), (5), (6), the results is shown in Table 3 and Fig. 8. The trend score $S_{NP}^{k}$ can be understood as the popularity of a topic. A higher trend score indicates that the topic is gaining more attention in research and highly popular in recent studies. The normalized score $S_{NTr}^{k}$ represents the proportional score of the topic. A higher normalized score implies that the topic occupies a larger proportion in the overall research landscape. In summary, the trend score $S_{NP}^{k}$ (popularity) reflects the temporal changes in the topic's popularity, while the normalized score $S_{NTr}^{k}$ (proportional score) indicates the overall proportion of the topic in the research literature. $P^{k}$ is the sum of the trend score and the normalized value. By examining these two metrics, this study could gain a more comprehensive understanding of the importance and development trends of a topic in precision agriculture research.

**Table 3:** Popularity ranking of topics.

Rank	Topic	$S_{NP}^{k}$	$S_{NTr}^{k}$	$P^{k}$	Rank	Topic	$S_{NP}^{k}$	$S_{NTr}^{k}$	$P^{k}$
1	Soil Management and Fertilization (T8)	0.43	1	1.43	20	UAVs and Drone Applications (T18)	0.38	0.49	0.87
2	Water Management and Irrigation (T10)	0.52	0.89	1.4	21	Chromatography and Pesticide Detection (T17)	0.33	0.52	0.85
3	Wireless Sensor Networks (T12)	0.52	0.59	1.11	22	Genetic and Molecular Analysis (T14)	0.33	0.52	0.85
4	Land Classification and Remote Sensing (T4)	0.32	0.79	1.1	23	Spray Technology and Application (T27)	0.35	0.5	0.85
5	Soil Erosion and Sediment Transport (T31)	0.45	0.63	1.08	24	IoT and Smart Agriculture (T2)	0.42	0.42	0.84
6	Hyperspectral Imaging and Spectral Analysis (T13)	0.34	0.72	1.06	25	Soil Spectroscopy and Analysis (T24)	0.28	0.49	0.77
7	Robotics and Autonomous Systems (T7)	0.34	0.69	1.03	26	Electrochemical Sensors and Nanotechnology (T16)	0.41	0.34	0.75
8	Water Pollution and Quality (T28)	0.43	0.59	1.02	27	Climate-Smart Agriculture and Adaptation (T9)	0.31	0.44	0.75
9	Digital Farming and Technology Adoption (T6)	0.52	0.49	1	28	Livestock Management (T5)	0.45	0.29	0.74
10	UAV and Aerial Imaging (T11)	0.45	0.55	1	29	Fog and Edge Computing in Agriculture (T37)	0.4	0.32	0.72
11	Seed and Yield Monitoring (T19)	0.34	0.62	0.96	30	Plant Disease Detection (T25)	0.39	0.31	0.7
12	Heavy Metals and Soil Pollution (T36)	0.52	0.43	0.95	31	IoT Security and Privacy (T26)	0.39	0.3	0.69
13	Aquaculture and Water Quality (T35)	0.58	0.35	0.93	32	LoRa and IoT Networks (T30)	0.39	0.29	0.68
14	Big Data Analytics in Agriculture (T29)	0.47	0.45	0.92	33	Deep Learning and Image Segmentation (T20)	0.22	0.31	0.53
15	Renewable Energy and Solar Power (T33)	0.53	0.39	0.92	34	Fruit Detection and Counting (T32)	0.23	0.3	0.53
16	Soil Conductivity and Moisture Measurement (T23)	0.35	0.56	0.91	35	Weed Identification and Control (T34)	0.22	0.31	0.53
17	Emissions and Carbon Management (T15)	0.31	0.59	0.9	36	Blockchain and Supply Chain (T21)	0.27	0.23	0.5
18	Machine Learning and Prediction (T1)	0.28	0.61	0.9	37	Disease Detection and Diagnosis (T3)	0.14	0.31	0.44
19	Image Processing and Weed Detection (T22)	0.33	0.57	0.89

As shown in Table 3, the topics Soil Management and Fertilization (T8), Water Management and Irrigation (T10), and Wireless Sensor Networks (T12) dominate. Soil Management and Fertilization (T8) and Water Management and Irrigation (T10) belong to the subfield of Soil and Water Management, while Wireless Sensor Networks (T12) falls under IoT Innovation in Agriculture. This result aligns with the trend analysis, particularly the notable upward trend in IoT Innovation in Agriculture observed in Fig. 7. Additionally, topics that are ranked in the middle tend to have either higher topic proportions or a balance of both metrics. Lower-ranked topics do not show significant differences in $S_{NTr}^{k}$ , and thus, rely more on $S_{NP}^{k}$.

### 3.3 Topic distribution over countries

**Fig. 9:**
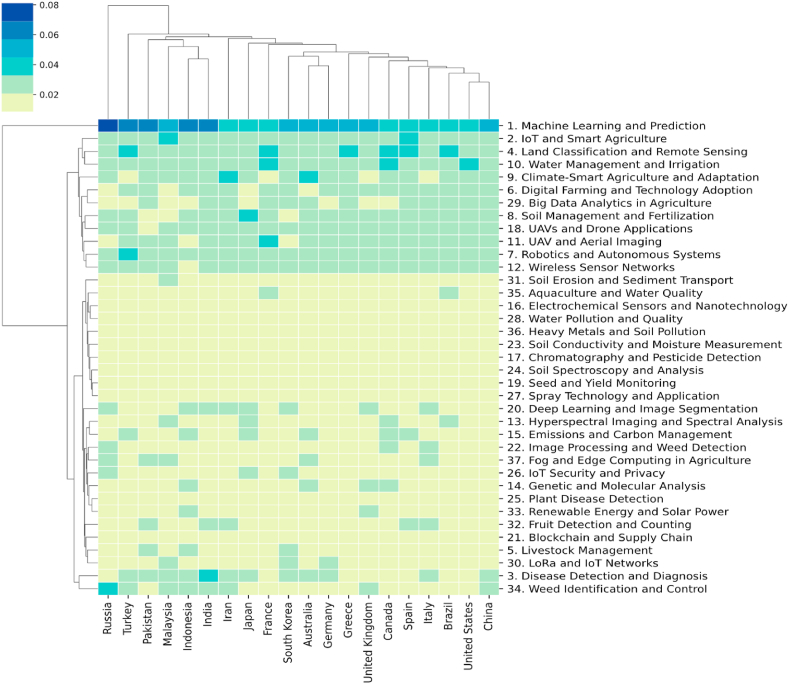
Topic distribution over countries.

This section calculates the topic distribution at the national level using Equation (7) and focuses on the top 20 countries by publication volume. These distributions are displayed in a heatmap in Fig. 9. In the heatmap, the columns represent research topics, while the rows represent countries. The relationship between a country and research topics is indicated by the probability of a topic appearing at the national level. Furthermore, the country-topic distribution resembles a matrix, where the values can be considered as the distance between a country and a topic. This suggests that clustering methods can be applied here. Therefore, in this study, the distances between publication sources and research topics were calculated using the Euclidean distance, and hierarchical clustering was performed using the average linkage method. The clustering results are displayed in the dendrograms on the left side and top of Fig. 9. From these results, it is possible to observe which topics each country focuses on, which topics are categorized together, and which countries tend to concentrate on similar types of research.

The clustering results from Fig. 9 indicate that certain countries grouped together often share close geographical relationships. For example, Malaysia, India, and Indonesia are clustered together due to their proximity. However, China, the United States, South Korea, Australia, and Germany are not neighboring countries but are still categorized into the same group. This phenomenon suggests that international collaborations often transcend geographical boundaries. From the heatmap of topic distribution at the national level, it is clear that the most prominent topics currently of interest to most countries are "Machine Learning and Prediction" and "IoT and Smart Agriculture." These topics are also distinctly categorized into a major group in the hierarchical clustering of topics.

**Fig. 10:**
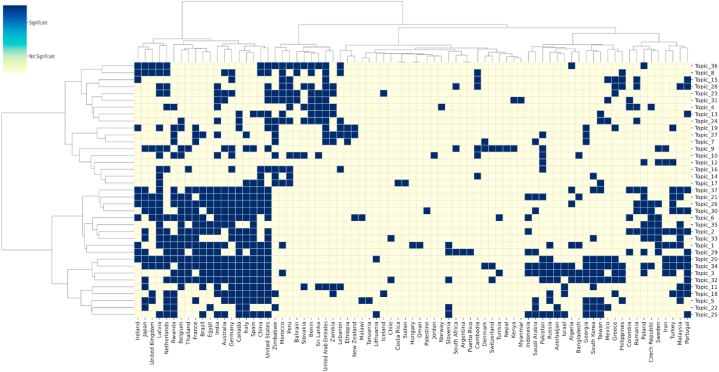
The hot and cold topics for each country.

The analysis of publication sources and topic distributions among different countries in Fig. 9 explores the variations and similarities in research themes across nations. However, this analysis is static and does not capture changes in topic distributions within countries over time. To address this, the study aggregated the topic distributions for each country using Equation (8). The aggregated results indicate that research themes in different countries evolve over time, although these changes are subtle. To further explore the dynamics of topic distributions in each country, this paper conducted a linear regression analysis on the time series of topic distributions, as illustrated in Equation (9). Following the analysis, each publication source is associated with a linear equation, $\varphi_{k}^{c,y}=a\times y+b$*,* for each of the 37 topics over time, $\varphi_{k}^{c,y}$ defined by Equation (8). Here, represents the proportion of a topic in a country c in a given year y, a and b are the slope and intercept of the equation, respectively. It is important to note that if the linear regression results for a topic are not significant, that topic is discarded (p≤0.05), and countries without any significant hot topics will not be featured in the visualizations. To enhance interpretability, hierarchical clustering was also applied to the results. The hot and cold topics for each publication source are depicted in Fig. 10.

Initial hierarchical clustering depicted in Fig. 10 reveals that China and the United States, Italy and Spain, as well as Germany and Australia, are grouped together. This phenomenon aligns with the results from Fig. 9, indicating not only a similarity in the topic focus of these countries but also a parallel trend in the rising popularity of certain topics. It is noteworthy that the topics of Weed Identification and Control (T34) and Disease Detection and Diagnosis (T3) rank among the top concerns in multiple countries/regions, including China, the United States, and Brazil. Conversely, Wireless Sensor Networks (T12) and Genetic and Molecular Analysis (T14) are less popular, appearing as cold topics in 68 and 67 countries, respectively. This trend suggests a global decline in interest towards shape representation (T12) and robotic manipulation (T14).

**Fig. 11:**
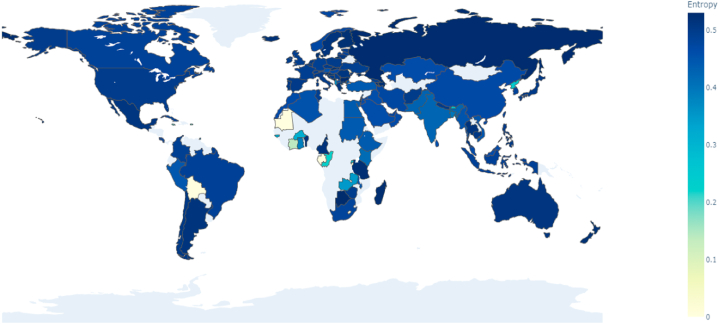
Worldwide map visualization displaying the spatial distribution of topic information entropy among nations.

Upon analyzing the distribution of topics across nations and the temperature of topics within each country, this study further employs Equation (10) to calculate the information entropy for each country. The resulting data elucidates the breadth of research content within the domain of precision agriculture for each nation. Information entropy measures the diversity and uncertainty of topic distribution; a higher entropy value indicates a more balanced spread across multiple research topics, reflecting greater diversity. Conversely, a lower entropy value suggests a more concentrated research focus, with less diversity. As shown in Fig. 11, Botswana exhibits the highest information entropy at 0.5307, closely followed by Slovenia (0.5306) and Montenegro (0.5304). Additionally, Finland, Sweden, and Denmark demonstrate high entropy values. Globally, high entropy is predominantly found in North America (e.g., Canada and the United States), Western Europe (e.g., the United Kingdom and Germany), and certain Asian countries (e.g., Japan and Singapore), indicating not only a broad scope but also an even distribution of agricultural research themes. In contrast, Eastern Europe, Africa, and some Middle Eastern countries, such as Iran and Saudi Arabia, display lower entropy values, suggesting a more limited and focused range of agricultural research topics in these regions.

### 3.4 Topic distribution over journals

**Fig. 12:**
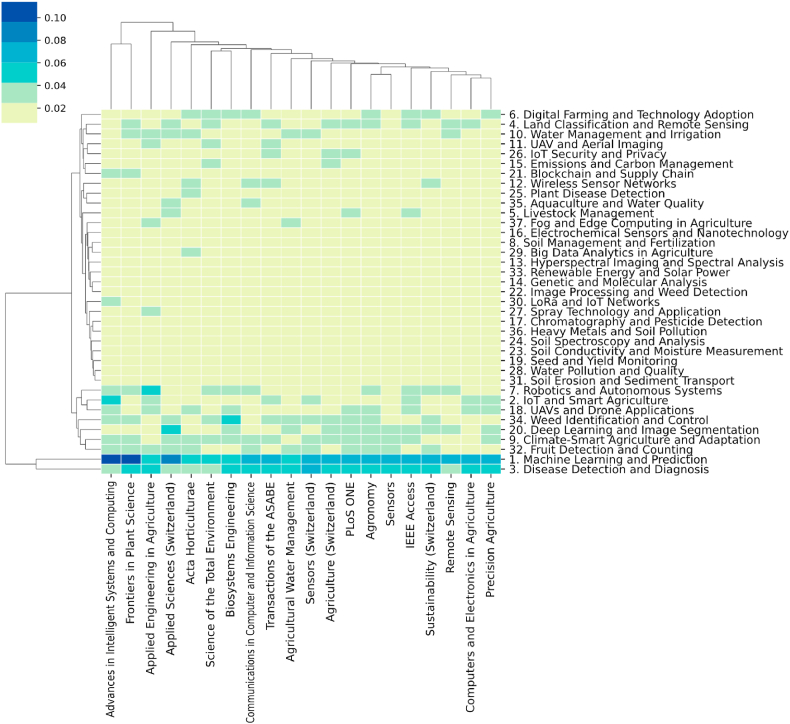
Topic distribution over journals.

This section calculates the topic distribution of publication sources through Equations (11), (12) and presents them in a heatmap within Fig. 12. Mirroring the country-level analysis of Section 3.3, this study selects the top 20 journals with the highest volume of published literature. In the realm of precision agriculture, most journals exhibit a dispersed array of research topics, not confined to a select few. However, a minority of journals demonstrate a certain concentration in their research themes. Notably, Computers and Electronics in Agriculture focuses on topics such as Machine Learning and Prediction (T1) and Disease Detection and Diagnosis (T3), indicating a keen interest in Data Analysis and Machine Learning. This trend is also prevalent in other journals, for instance, Precision Agriculture and Frontiers in Plant Science. Additionally, Robotics and Autonomous Systems (T7), Climate-Smart Agriculture and Adaptation (T9), and Weed Identification and Control (T34) are focal points for Science of the Total Environment.

**Fig. 13:**
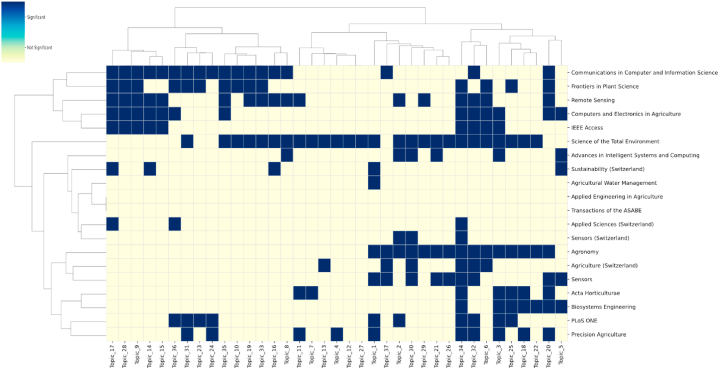
Hot and cold topics distribution over journals.

In addition to static analysis of topic distributions, a dynamic analysis of the topic spread within the top 20 ranked journals is also imperative. Utilizing the linear regression model introduced in Section 3.3, this section examines the time series of the journals’ topic distributions. Following the same criteria for selection, the hot and cold topics of the top 20 journals are ultimately listed in Fig. 13.

An examination of Fig. 13 at the journal level reveals the varying degrees of interest in different topics across publications. The three journals with the hottest topics are: Science of the Total Environment, with a significant count of 26. This indicates that out of the total 37 topics, 26 are considered hot topics within the Science of the Total Environment journal. Following closely are Communications in Computer and Information Science, with 18 hot topics, and Remote Sensing, with 17. This suggests that these journals not only have a multitude of hot topics but also a broad range. Communications in Computer and Information Science and Frontiers in Plant Science share a similar array of hot topics and are thus clustered together. A similar pattern is observed with Computers and Electronics in Agriculture and IEEE Access. At the topic level, Fruit Detection and Counting (T32) and Weed Identification and Control (T34) emerge as hot topics in most journals, underscoring their popularity within the publications. This trend is also evident in the clustering of topics at the topic level.

### 3.5 BERTopic evaluation

This research involved analyzing a textual corpus of literature on precision agriculture, comparing the BERTopic and LDA models in terms of topic coherence (TC) and topic diversity (TD) using Equations (13), (14), (15). The findings indicated that BERTopic achieved a TC value of 0.7, significantly higher than LDA's 0.27. This suggests that the BERTopic model provides better topic consistency, generating more coherent and clear themes. This improvement may stem from BERTopic's advanced methods in text embedding and clustering techniques, which more effectively capture semantic relationships between documents. While LDA's TD value stands at 0.65, slightly above BERTopic's 0.6, indicating a slight edge in topic diversity, this diversity can result in overlapping and ambiguous themes, reducing their interpretability and clarity.

## 4 Discussion

### 4.1 Comparison with previous studies

From a methodological perspective, this study utilizes text mining models to extract topics from article abstracts, keywords, and titles, focusing on the distribution and evolution of topics within the precision agriculture domain. Specifically, BERTopic, a model based on the transformer architecture, is employed for its enhanced topic refinement and diversity. Comparing to other traditional topic modeling, Latent Dirichlet Allocation (LDA), well-grounded in theory and widely used, offers a generative model for data processes but struggles with high computational complexity, hyperparameter sensitivity, and less intuitive interpretability [23,24]. Non-negative Matrix Factorization (NMF) is valued for its straightforward interpretation, computational efficiency, and sparse solutions, which aid thematic clarity, yet it is prone to local optima and lacks a generative model [25]. In contrast, BERTopic harnesses transformer-based embeddings like BERT to capture rich semantic nuances, providing high flexibility and clear topic structure through hierarchical clustering. However, it requires significant computational resources, is complex, and depends on pre-trained model quality [26]. Despite these advancements, topic modeling approaches inherently have limitations. For example, all models, including BERTopic, can be affected by biases introduced during data preprocessing and selection, as well as the difficulty of accurately interpreting the abstract nature of topics. To address these issues, we implemented a rigorous data preprocessing pipeline to minimize noise and ensure data quality. Additionally, we conducted extensive parameter tuning and validation to optimize the model's performance and enhance the reliability of our results.

Additionally, unlike prior studies, this research presents a novel comprehensive popularity index that integrates trend intensity and topic proportion. This approach capitalizes on the scoring benefits of topic model outputs to identify emerging topics. Moreover, it examines the distribution characteristics of academic knowledge across various dimensions (e.g., country, journal) and delves into the intricacies of topic trends at a granular level. These elements have been underexplored in earlier research. This comprehensive approach is challenging to achieve with a single model alone, highlighting the strength of combining advanced NLP technologies with traditional methodologies.

On the result analysis, previous scholars have primarily focused on specific areas, such as deep learning in agriculture [27], hyperspectral imaging in precision agriculture [28], or analyses centered on certain countries, such as the United States [1]. In contrast, this study analyzes the entire field of precision agriculture based on sources of professional knowledge. It emphasizes the trends in topic distribution and identifies the characteristics of topic distribution at both the publication source and national levels. This approach provides a more nuanced analysis compared to previous studies, enhancing the depth and breadth of the research.

### 4.2 Main findings of this study

#### 4.2.1 Topic finding

##### 4.2.1.1 Data Analysis and Machine Learning

By analyzing topic distribution and subdividing the field, deeper research characteristics in precision agriculture can be identified. For example, the significant focus on Machine Learning and Prediction (T1) and the related topics like Deep Learning and Image Segmentation (T20) and Disease Detection and Diagnosis (T3) imply that machine learning is extensively used for predictive analytics and disease diagnostics in agriculture. This is consistent with previous research that emphasizes using sophisticated algorithms for identifying patterns and making predictions to enhance agricultural productivity and disease management [29]. A representative case study includes the application of deep learning techniques for real-time disease detection in crops, showcasing practical benefits in early disease intervention and yield optimization [30].

Similarly, the topics of Fruit Detection and Counting (T32) and Weed Detection and Identification, Weed Identification and Control (T22 and T34) highlight the application of machine learning in detecting and managing crops and weeds. These advancements suggest a shift towards automating labor-intensive tasks, thereby reducing labor costs and increasing precision in crop management [31]. The use of image-based machine learning models for identifying fruit maturity stages [32] and detecting weed infestations [33] exemplifies this trend. Such applications not only improve accuracy in agricultural practices but also contribute to sustainable farming by optimizing resource use and minimizing chemical inputs.

The significance of these topics lies in their potential to revolutionize precision agriculture by providing actionable insights and automating complex tasks. This shift towards data-driven decision-making and automation is poised to address key challenges in agriculture, such as labor shortages, disease management, and efficient resource use, ultimately leading to increased productivity and sustainability.

##### 4.2.1.2 IoT Innovation in Agriculture

The detailed analysis of topic distribution highlights significant advancements in IoT Innovation within agriculture. Key topics such as IoT and Smart Agriculture (T2), IoT Security and Privacy (T26), and LoRa and IoT Networks (T30) demonstrate the increasing integration of IoT devices in farming practices. These technologies are essential for real-time monitoring and data collection, which enhance the efficiency and security of agricultural operations [34]. The use of IoT solutions enables precise control over various farming processes, contributing to optimized resource utilization and improved crop management [35].

Furthermore, the inclusion of topics like Wireless Sensor Networks (T12) and Fog and Edge Computing in Agriculture (T37) indicates a growing adoption of edge computing technologies. These technologies allow data to be processed closer to the source, thereby improving response times and reducing bandwidth usage. This is particularly valuable in remote agricultural settings where connectivity can be a challenge [36]. The deployment of edge computing infrastructures supports timely decision-making and facilitates more effective farm management by providing real-time insights into environmental conditions and crop health [37].

These advancements in IoT and related technologies are transforming the agricultural landscape, offering enhanced precision, efficiency, and security in agricultural operations. The continued innovation in this area is expected to drive further improvements in the sustainability and productivity of farming practices.

##### 4.2.1.3 UAVs and remote sensing

The analysis highlights the critical role of UAVs and Remote Sensing in precision agriculture, as evidenced by topics such as UAV and Aerial Imaging (T11) and Drone Applications (T18). These topics demonstrate the extensive use of drones for monitoring and data collection, facilitating detailed and accurate assessments of agricultural fields. Drones equipped with advanced imaging technologies are employed to capture high-resolution aerial images, which are crucial for identifying crop health issues, optimizing irrigation, and managing pests [38].

Moreover, topics like Land Classification and Remote Sensing (T4) and Hyperspectral Imaging and Spectral Analysis (T13) showcase the application of sophisticated techniques for analyzing land and crop conditions. These technologies enable the precise classification of land types and the detection of subtle changes in crop health that are not visible to the naked eye. Hyperspectral imaging allows for the detailed analysis of crop physiology and stress factors, providing actionable insights for farm management [39].

These advanced remote sensing techniques are pivotal in precision agriculture, offering real-time data and analytics that enhance decision-making processes. By utilizing UAVs and sophisticated imaging technologies, farmers can implement more targeted interventions, ultimately leading to improved crop yields, efficient resource use, and sustainable farming practices. The integration of these technologies underscores a shift towards data-driven agriculture, where real-time information plays a key role in optimizing agricultural operations.

##### 4.2.1.4 Soil and Water Management

The domain of Soil and Water Management is crucial in precision agriculture, as indicated by the emphasis on topics such as Soil Management and Fertilization (T8), Soil Conductivity and Moisture Measurement (T23), and Water Management and Irrigation (T10). These topics highlight the importance of optimizing soil health and efficient water use, which are key to enhancing crop productivity and sustainability. Advanced techniques in soil conductivity and moisture measurement allow for precise monitoring and management of soil conditions, ensuring that crops receive adequate nutrients and water.

Additionally, topics like Soil Erosion and Sediment Transport (T31) and Water Pollution and Quality (T28) underscore significant environmental concerns. They reflect the growing need for sustainable practices that effectively manage natural resources and mitigate negative environmental impacts. These topics emphasize the importance of preventing soil degradation and water contamination, which are critical for maintaining ecosystem health and agricultural productivity [40].

Incorporating sustainable soil and water management practices not only addresses environmental challenges but also contributes to the long-term viability of farming operations. By focusing on these areas, precision agriculture can enhance resource efficiency, reduce environmental footprint, and promote the sustainable use of natural resources, ultimately leading to more resilient agricultural systems [41].

##### 4.2.1.5 Crop and Pest Management

The focus on Crop and Pest Management is underscored by topics such as Plant Disease Detection (T25), Spray Technology and Application (T27), and Seed and Yield Monitoring (T19). These topics illustrate the integration of advanced technologies in monitoring crop health, controlling pests, and optimizing yields. Plant Disease Detection technologies enable early identification and management of diseases, which is crucial for minimizing crop losses and ensuring healthy harvests [42]. Spray Technology and Application highlight the precision in applying pesticides and nutrients, thereby improving efficiency and reducing the environmental impact [43].

Additionally, Seed and Yield Monitoring provides critical data for evaluating crop performance and yield outcomes [44]. This data-driven approach supports informed decision-making and allows for adjustments in real-time to optimize productivity. The use of these technologies reflects an integrated approach to agricultural management that combines various tools and methods to enhance crop productivity and sustainability. This holistic strategy not only improves the effectiveness of pest and disease management but also supports sustainable agricultural practices by optimizing input usage and minimizing waste.

##### 4.2.1.6 Livestock and Aquaculture

The focus on Livestock and Aquaculture is evident through topics like Livestock Management (T5) and Aquaculture and Water Quality (T35). These areas reflect the growing incorporation of AI and advanced technologies in managing animal health and optimizing aquaculture environments. In livestock management, AI-driven systems enhance the monitoring and analysis of animal health, improving disease prevention, welfare, and productivity [45]. These technologies facilitate precision in feeding, breeding, and overall herd management, leading to more efficient and sustainable livestock production.

In aquaculture, the emphasis on water quality and environmental monitoring showcases the importance of maintaining optimal conditions for aquatic life. Technologies in this domain enable real-time monitoring of water parameters, ensuring the health and growth of aquaculture species. This not only helps in preventing diseases but also promotes sustainable practices by optimizing the use of resources and minimizing environmental impacts [46].

The integration of these innovations contributes significantly to improving efficiency and sustainability in livestock and aquaculture practices. By leveraging AI and technology, producers can achieve higher productivity, better animal welfare, and reduced ecological footprints, aligning with the goals of sustainable agriculture and food security.

##### 4.2.1.7 Sustainable Agriculture

Sustainable Agriculture is highlighted as a critical focus area, with topics such as Climate-Smart Agriculture and Adaptation (T9), Emissions and Carbon Management (T15), and Renewable Energy and Solar Power (T33) indicating a strong emphasis on promoting environmentally friendly practices. These topics underscore the importance of adapting agricultural practices to changing climate conditions and reducing the carbon footprint of farming activities. Efforts in climate-smart agriculture aim to enhance resilience [47], while the adoption of renewable energy sources like solar power contributes to reducing dependency on fossil fuels and mitigating greenhouse gas emissions [48].

Additionally, the focus on Electrochemical Sensors and Nanotechnology (T16) and Heavy Metals and Soil Pollution (T36) reflects the application of advanced technologies to monitor and manage environmental impacts. These technologies play a crucial role in detecting pollutants and assessing soil health, enabling more precise and effective responses to environmental challenges. The use of electrochemical sensors and nanotechnology helps in identifying and mitigating the presence of heavy metals and other contaminants, thereby protecting soil quality and ensuring safe food production [49].

These advancements in sustainable agriculture not only promote environmental stewardship but also support the long-term viability of agricultural systems. By integrating innovative technologies and sustainable practices, agriculture can become more resilient and less harmful to the environment, aligning with global goals for sustainable development and climate action.

##### 4.2.1.8 Agricultural Technology and Innovation

Agricultural Technology and Innovation encompasses a wide array of advancements, as demonstrated by topics such as Digital Farming and Technology Adoption (T6), Robotics and Autonomous Systems (T7), and Blockchain and Supply Chain (T21). These topics illustrate the rapid incorporation of new technologies to enhance farming efficiency and productivity. Digital farming technologies facilitate data-driven decision-making [50], while robotics and autonomous systems are revolutionizing field operations, from planting to harvesting, by automating labor-intensive tasks [51]. The application of blockchain technology in supply chain management offers improved traceability, transparency, and security, which are essential for maintaining the integrity of agricultural products [52].

Furthermore, topics like Genetic and Molecular Analysis (T14) and Chromatography and Pesticide Detection (T17) highlight the significant role of biotechnology and advanced analytical methods in agriculture. These technologies are pivotal in improving crop quality and safety. Genetic and molecular analysis techniques enable the development of crop varieties with desirable traits such as disease resistance and enhanced nutritional value [53]. Meanwhile, chromatography and pesticide detection methods ensure that agricultural products meet safety standards by accurately identifying and quantifying pesticide residues [54].

These technological advancements are integral to modernizing agricultural practices, enhancing food safety, and ensuring sustainable food production. By embracing innovation, the agricultural sector can achieve greater efficiency, reduce environmental impact, and provide safer, higher-quality products to consumers.

#### 4.2.2 Topic trend

The BERTopic algorithm surfaced 37 discernible topics in the area of precision agriculture, aggregating into eight overarching categories: Data Analysis and Machine Learning, IoT Innovation in Agriculture, UAVs and Remote Sensing, Soil and Water Management, Crop and Pest Management, Livestock and Aquaculture, Sustainable Agriculture, Agricultural Technology and Innovation. These categories can be regarded as 8 subfields within the precision agriculture domain, with topics representing specific research directions within these subfields.

The topic trend illustrates a clear evolution in the research focus within the field of precision agriculture. Initially, there was a high interest in Agricultural Technology and Innovation, reflecting early enthusiasm for technological advancements. However, the declining trend may indicate that while technology adoption remains crucial, the novelty may have worn off, leading to a relative decrease in focus.

Conversely, the growing interest in Crop and Pest Management suggests a heightened emphasis on optimizing crop production and protecting plants from pests, possibly driven by the increasing demand for food security and sustainable agricultural practices [55]. The rise in IoT Innovation in recent years is particularly surprising, especially since traditional areas like Data Analysis and Machine Learning continue to dominate the field. The sudden increase in IoT applications in agriculture, particularly since 2022, reflects a rapid adoption of smart technologies, likely influenced by the broader advancements in artificial intelligence and digital transformation [35]. This trend enhances real-time monitoring and decision-making capabilities, highlighting a significant shift towards more interconnected and intelligent agricultural systems.

The relatively steady trends in Data Analysis and Machine Learning and Soil and Water Management suggest ongoing importance but without significant new surges, indicating established methodologies and stable research interest. The moderate and steady rise in Sustainable Agriculture points towards a growing awareness and implementation of environmentally friendly practices [56].

The fluctuating yet stable trend in UAVs and Remote Sensing reflects its established role in aerial monitoring and data collection, while the moderate interest in Livestock and Aquaculture suggests niche but vital contributions to the field.

Overall, the topic trend highlights how research priorities in precision agriculture have shifted towards integrating advanced technologies like IoT and machine learning, emphasizing sustainable practices, and managing crops and pests more effectively. The unexpected rapid growth in IoT innovation underscores the need for academia and industry to recognize and capitalize on this trend, aligning with the broader movement towards precision and sustainability in agriculture. This shift aims for efficient resource use, enhanced productivity, and reduced environmental impact [57], demonstrating a dynamic and evolving landscape in agricultural research and practice.

#### 4.2.3 Topic distribution over countries

The global landscape of precision agriculture research is shaped by a complex interplay of economic, geographical, and technological factors that influence the focus of scientific inquiry in various countries. Key economic priorities and the scale of the agricultural sector significantly drive research topics in countries like China and the United States, where there is a heightened focus on advanced technologies such as Machine Learning, Prediction, and IoT in agriculture. These technologies are prioritized due to their potential to enhance agricultural productivity and efficiency, which is crucial given the substantial economic contributions of agriculture in these regions [58].

Geographical and climatic conditions also play a critical role in determining research priorities. For instance, the focus on Weed Identification and Control, and Disease Detection and Diagnosis in countries like Brazil and the United States can be linked to their diverse climatic conditions, which affect pest prevalence and disease spread. Addressing these challenges is essential for maintaining crop health and productivity, thus receiving significant research attention [59].

The infrastructure for technology and levels of investment also influences the prominence of certain research topics. Countries like Germany and Australia, with robust technological infrastructures and substantial R&D investments, are leaders in integrating sophisticated agricultural technologies such as wireless sensor networks [60]. Furthermore, policy and governmental support, particularly in European countries like Italy and Spain, steer research directions through aligned agricultural policies and subsidies [61], reflecting a regional consensus on priority research areas.

The analysis also reveals that international collaborations and scientific exchanges transcend geographical boundaries, as evidenced by the clustering of non-neighboring countries like China with the United States and Germany with Australia in hierarchical clustering. This suggests a global synchronization of research efforts facilitated by shared scientific goals and international platforms.

Information entropy values further elucidate the breadth and diversity of research within countries. High entropy values in countries like Canada, the UK, and Japan indicate a diverse research portfolio, while lower values in Eastern Europe and parts of the Middle East suggest a more concentrated focus on specific agricultural challenges. This diversity is indicative of a comprehensive approach to tackling agricultural issues, fostering innovation across multiple fronts.

In summary, the dynamics of precision agriculture research are shaped by a myriad of factors including economic significance, geographical and climatic challenges, technological capability, and policy frameworks. Understanding these influences helps in mapping current research focuses and predicting future trends and collaborations in the field of global agricultural research. The analysis highlights a shared focus on integrating advanced technologies and addressing environmental challenges, which aligns closely with the topic trends identified in the study. This convergence emphasizes a collective global effort towards optimizing agricultural practices through precision agriculture, aiming for sustainable and efficient resource use.

#### 4.2.4 Topic distribution over journals

The analysis of topic distributions within top journals in precision agriculture reveals a nuanced landscape shaped by various factors that influence the concentration of research interests. Journals such as "Computers and Electronics in Agriculture" and "Precision Agriculture" demonstrate a specific focus on advanced topics like Machine Learning and Prediction due to their explicit editorial scope that aligns with technological advancements in agriculture. This alignment not only attracts submissions in these cutting-edge areas but also reflects the journals' commitment to advancing specific segments of agricultural research.

Furthermore, the diversity of hot topics in journals such as "Science of the Total Environment" underscores their interdisciplinary nature, allowing for a broad coverage of issues from Climate-Smart Agriculture to Weed Identification and Control. This breadth is indicative of a strategic editorial choice to embrace a wide array of environmental and agricultural challenges, catering to a diverse academic audience and reflecting global concerns in sustainability and efficiency.

In summary, the primary focus of these journals on topics related to Data Analysis and Machine Learning, particularly Machine Learning and Prediction, mirrors the global trends observed in precision agriculture research. This alignment highlights a shared interest across academic, national, and journalistic landscapes in leveraging data-driven approaches and machine learning technologies. The concentration of research efforts in these areas underscores the importance of advancing precision agriculture through sophisticated data analysis and predictive techniques, as reflected in the distribution of research interests within these leading journals.

## 5 Conclusion and limitation

This study employed the BERTopic algorithm to examine the distribution and evolution of research topics in precision agriculture. It identified significant trends, notably the increasing focus on IoT and data-driven technologies, while also recognizing the continued relevance of traditional agricultural practices.

However, the analysis was confined to article abstracts, keywords, and titles, which may not capture the full depth of insights available in the full texts. The study primarily addressed static and evolving topic distributions but could be enriched with advanced time-series analyses to better predict future trends. There is also a potential under representation of research from less technologically advanced regions. Additionally, emerging topics may not be fully captured due to the temporal limitations of the dataset.

Future research should include a broader range of publications and geographic areas, incorporate qualitative analyses, and consider engaging with industry stakeholders to enhance the practical relevance of the findings. Continuous updates to the topic model are essential for accurately identifying new trends.

## Funding statement

This research did not receive any specific grant from funding agencies in the public, commercial, or not-for-profit sectors.

## Data availability statement

Data will be made available on request.

## Additional information

No additional information is available for this paper.

## CRediT authorship contribution statement

**Yang Liu:** Writing – review & editing, Writing – original draft, Visualization, Supervision, Software, Methodology, Formal analysis, Data curation. **Fanghao Wan:** Writing – review & editing, Supervision.

## Declaration of competing interest

The authors declare that they have no known competing financial interests or personal relationships that could have appeared to influence the work reported in this paper.
